# How Alpha Linolenic Acid May Sustain Blood–Brain Barrier Integrity and Boost Brain Resilience against Alzheimer’s Disease

**DOI:** 10.3390/nu14235091

**Published:** 2022-11-30

**Authors:** Alicia Leikin-Frenkel, Michal Schnaider Beeri, Itzik Cooper

**Affiliations:** 1 Bert Strassburger Lipid Center, Sheba Medical Center, Tel-Hashomer 52621, Israel; 2 Sackler Faculty of Medicine, Tel-Aviv University, Tel-Aviv 69978, Israel; 3 The Joseph Sagol Neuroscience Center, Sheba Medical Center, Ramat-Gan 52621, Israel; 4 Department of Psychiatry, The Icahn School of Medicine at Mount Sinai, New York, NY 10029, USA; 5 School of Psychology, The Reichman University (IDC), Herzliya 4610101, Israel

**Keywords:** cardiocerebrovascular diseases, fatty acids, alpha linolenic acid, Alzheimer’s disease, Alzheimer’s dementia, vascular cognitive impairment, blood–brain barrier

## Abstract

Cognitive decline, the primary clinical phenotype of Alzheimer’s disease (AD), is currently attributed mainly to amyloid and tau protein deposits. However, a growing body of evidence is converging on brain lipids, and blood–brain barrier (BBB) dysfunction, as crucial players involved in AD development. The critical role of lipids metabolism in the brain and its vascular barrier, and its constant modifications particularly throughout AD development, warrants investigation of brain lipid metabolism as a high value therapeutic target. Yet, there is limited knowledge on the biochemical and structural roles of lipids in BBB functionality in AD. Within this framework, we hypothesize that the ApoE4 genotype, strongly linked to AD risk and progression, may be related to altered fatty acids composition in the BBB. Interestingly, alpha linolenic acid (ALA), the precursor of the majoritarian brain component docosahexaenoic acid (DHA), emerges as a potential novel brain savior, acting via BBB functional improvements, and this may be primarily relevant to ApoE4 carriers.

## 1. Introduction

Treatment for neurodegeneration is practically non-existent. A major reason relates to the double-edged sword role of the blood–brain barrier (BBB). It both restricts the entry of potential therapeutics and is impaired already in the earliest stages of neurodegeneration [1]. The BBB is a crucial player in maintaining brain health as it prevents the entrance of unfavorable molecules and removes physiological waste from the brain’s parenchyma, if the BBB’s clearance machinery operates properly [2]. The *question of whether lipids and specifically fatty acid (FA) composition can regulate BBB function* has yet to be elucidated. Transport of lipids to the brain is mediated by the major facilitator superfamily domain containing 2A (Mfsd2a) lining the BBB [3,4]. The results of lipidomic profiling of brain capillaries, point to lipid composition of brain endothelial cells as a key regulator for BBB lipid transport [5]. Therefore, the lipid disarray related to BBB malfunction- directly implicated in numerous brain diseases, primarily neurodegeneration—becomes a major target for potential new treatments [6].

Worldwide, the number of people with dementia is about 55 million and this number is expected to double every 20 years [7], making dementia a major epidemic of this century. However, currently approved drugs primarily treat symptoms and have limited impact on the course of disease. There are over 140 drugs in the current AD drug development pipeline. Disease-modifying therapies represent 83.2% of the candidate treatments. Drugs for prevention of dementia however, are scarce [8]. Yet, modifiable risk factors for dementia, such as the diet, consistently linked to slower rate of cognitive decline, lend promise for prevention or delay of the disease [9]. Specifically in the context of lipids, lipid supplementation have been shown to improve health metrics such as insulin resistance, fatty liver and BBB integrity [10,11,12], and to extend life span, modulating the aging processes [13]. Specific lipid-based nutrients, such as the n-3 polyunsaturated fatty acids (PUFA) have been linked to better cognitive health due to their anti-inflammatory and synaptogenic properties [14]. Nevertheless, the results of dietary interventions on brain disease remain inconclusive [15] and the data establishing causative effects of nutrition on cognitive health remain uncertain. Importantly, populations with specific genetic backgrounds may more strongly benefit from dietary interventions. Specifically, recent discoveries show that in Alzheimer’s disease (AD), ApoE4 differentially impacts inflammatory pathways, lipid metabolism and BBB integrity [16]. There is initial evidence suggesting that among ApoE4 genotype carriers, who are at high AD risk, precision nutrition targeting metabolic pathways altered by ApoE4 provides a tool for the potential prevention of disease [16,17].

In this commentary, we briefly present the impact of FA on brain and BBB function in AD. In particular, we introduce the potential beneficial effects of the essential n-3 alpha linolenic acid [ALA, 18:3(n-3)].

## 2. The Blood–Brain Barrier (BBB)

The BBB is a selective interphase between systemic blood and brain parenchyma with brain endothelial cells as its principal cellular component. Its structural and functional stability is critical to keep a healthy brain. The brain blood capillaries are different from peripheral blood vessels due to the presence of tight junctions, lack of fenestrations, high number of mitochondria, restricted endocytosis and presence of unique transporters [18]. Pericytes and astrocytes are major cellular components of the neurovascular unit that constantly regulate barrier function in health and diseases [2]. The main brain endothelial cells biochemical structural components include (besides proteins) phospholipids, sphyngolipids and cholesterol, inserted in the membrane bilayer. Amphipatic phospholipids face the outer and inner aqueous environments thanks to their molecular hydrophilic groups whereas the hydrophobic esterified FA provide the lipophilic characteristics that allow the passage of some hydrophobic solutes across the BBB [19]. The essential n-3 ALA and LA [18:2(n-6)] FA, part of complex lipids, originate solely from the diet and are the source of metabolic PUFA products like docosahexaenoic [DHA, 22:6 (n-3)] and arachidonic (AA) acids [20] which participate in membrane remodeling. Besides, bodily synthesized saturated and monounsaturated FA (MUFA), contribute to the complex lipids properties, and to healthy brain structure and functioning [21]. The quality of FA transported through the BBB, which become critical elements of the membrane of all brain cells, will determine its physicochemical properties thereby influencing cells membrane fluidity, as well as receptors and transporters activities including those expressed in brain endothelial cells lining the BBB [22,23]. Although lipids are core molecular and cellular components mediating BBB function, research on BBB lipid composition and metabolism is rare. Conversely, research on brain lipids is abundant but, even when lipid alterations can be linked to the onset of age-related neurodegenerative diseases, evidence on the role of dietary FA have been restricted mostly to DHA. Recently we have shown that ALA, a metabolic precursor of DHA and n-3 PUFA, regulate the transport and metabolism of lipids in brain vasculature from neonatal stages to adulthood [24]. The gap in knowledge of the impact of essential ALA on the BBB structure, function, and brain lipid metabolism is a latent scientific drawback to overturn.

## 3. Lipids and BBB Function

The capillaries in brain provide the largest endothelial surface for molecular interchange between blood and brain [25]. Even though the brain is composed of nearly 60% of lipids, their role and, particularly their metabolism remains poorly understood [26]. A unique lipid composition of brain endothelial cells underlies BBB function and also influence the brain lipid supply of n-3 FA from peripheral circulation [5]. Lower transport of DHA, the main FA component of the brain [27], may be due to an altered BBB lipid composition and its associated lipophilic properties, which may lead to membrane disintegration and deficient Mfsd2a-mediated lipid transport. Moreover, in ApoE4 carriers, the deficient cholesterol transport from astrocytes to brain endothelial cells [28] may contribute to detrimental membrane lipid modifications in the BBB. High levels of DHA in brain vasculature can result from ALA metabolic transformation to DHA [24]. ALA effects are beneficial to the BBB lipid structure and subsequent function, which support our hypothesis that ALA may improve the BBB deterioration and the reduced FA transport associated with AD [24]. Alterations in the BBB membrane components have been shown to be effective in protecting its functional properties [25]. Interestingly, brain endothelial cells express key enzymes of de novo FA synthesis namely acetyl-CoA carboxylase and FA synthase [29], which are known to be regulated by dietary FA [30]. For all of the above reasons, we propose that repairing the BBB with the right FA, namely ALA, could reverse its structural and functional disarray in brain diseases in which BBB dysfunction is involved.

## 4. BBB Breakdown in AD from a Lipid Perspective

Growing evidence points to brain DHA reduced levels [31,32] in phospholipids and BBB breakdown as underlying molecular alterations of human cognitive impairment, including the early clinical stages of AD, even before amyloid deposition [1,33].

Neuroimaging studies in individuals with early AD as well as histological images of postmortem tissue have shown BBB breakdown in different brain areas [34]. Increased CNS permeability, reflecting loss of cerebrovascular integrity has also been shown, before dementia is ascertained [34]. For the cell membrane to keep normal function properly, it must maintain a balance between fluidity, which is influenced by the phospholipid head groups and FA components, and movement of proteins and lipids within the membrane, without compromising membrane integrity and allowing substances to leak into or out of the cell. These membrane characteristics have direct influence on the function of receptors and transporters expressed in brain endothelial cells, which have a crucial role in AD pathophysiology [35,36]. Human [37] and animal [38] studies indicate that n-3 FA components of the BBB, associated with dietary manipulation and aging [39], promote integrity and prevent its disruption. Importantly, a direct functional role of DHA has been demonstrated, following its Mfsd2a-mediated transport through the luminal side of brain endothelial cells, in suppressing the caveolae transcytosis and thereby maintaining BBB integrity [5]. It has also been shown that DHA confers long-term protection against ischemic brain damage through multiple mechanisms, including suppression of inflammatory responses, decrease in oxidative stress and stimulation of angiogenesis and neurogenesis [37]. DHA has also been associated with the decrease of Aβ deposition in animals [40], which combined with neuroinflammation, lead to BBB dysfunction [41]. We propose that, by conversion to DHA, ALA– the natural substrate in the DHA metabolic pathway– may produce the DHA beneficial effects on BBB and brain health. The following sections provide the rationale for this hypothesis.

## 5. Fatty Acids Role in Brain Health

Essential fatty acids (EFA) LA and ALA are precursors of the important brain components arachidonic acid [AA, 20:4(n-6)] and DHA (n-3), respectively, through processes of desaturation and elongation. The genes encoding the enzymes involved in those pathways are highly expressed in liver and brain [42,43]. Importantly, age related changes of FA desaturase activities have been described, including the affinity for its substrates which is higher for ALA than for LA later in life [44].

AA has been shown to have neurotoxic effects [45]. AA is also a precursor of various bioactive molecules including prostaglandins, such as PGE2 which has been implicated in greater BBB permeability [45]. Conversely, it has been shown that DHA is the precursor of enzyme-derived, neuroprotective docosanoids in brain [45]. Thus, the increase of DHA from ALA enrichment rather than AA from LA, may contribute to the prevention of BBB leakage [46].

DHA is notoriously decreased in the AD human brain [31], and so is the BBB function responsible for its transport. DHA brain levels seem to be controlled by the Mfsd2a transporter expressed on brain endothelial cells [4,47]. Importantly, a particular membrane FAs composition determines the optimal Δ6 desaturase activity for which they act as regulatory sensors [48]. These data highlight the need to further elucidate the inter-relationships between FA composition, BBB and brain health. Disentangling the etiology and chronology of brain lipid modifications in the BBB and particularly in AD would greatly broaden the landscape of potential preventive/therapeutic targets.

## 6. Dietary Fatty Acids

The essential ALA n-3 and LA n-6 FA, source of the metabolic PUFA DHA and AA, respectively, come from the diet. The brain is highly enriched in long-chain DHA and AA as structural components of neuronal membranes. These FA are precursors of lipid-derived prostaglandins and resolvins. When derived from DHA, they contribute to reverse inflammatory and other neurodegenerative processes [49]. Neural illnesses are believed to benefit from consumption of DHA and eicosapentaenoic acid [EPA, 20:5(n-3)] [50,51]. Surprisingly, whereas dietary maritime DHA supply has been regarded as the main efficient source of brain DHA [52], the ability of its metabolic precursor, terrestrial ALA, to support brain DHA (as well as EPA), has rarely been examined. Recent studies from our group and others [24,53] have begun to indicate, however, that ALA enrichment in adult diets and in maternal diets during fetal development and weaning: (1) enhances Mfsd2a expression in brain vasculature and (2) improves cognitive functions in adult offspring, compared to control diet. Weaning and adult offspring of ALA-fed dames have higher brain DHA levels and higher expression of the FA transporter Mfsd2a indicating the outstanding ALA ability to enable higher DHA levels and improved BBB transport [24]. Thus, we suggest that increased DHA, following ALA enrichment, may restore the BBB phospholipids FA composition and consequently enhance its structure and functional stability. Increased DHA brain accretion through enhanced Mfsd2a transport mechanism in AD and other neurodegenerative diseases, may lead, eventually, to improved memory.

## 7. Fatty Acid Metabolism

The conversion of essential ALA and LA to PUFA occurs via the same elongase and Δ6 desaturase enzymes in the metabolic pathway. Higher ALA levels usually overcome the LA substrate competition, allowing for the production of higher n-3 than n-6 PUFA metabolites [54]. Importantly, ∆6-desaturase expression is retro inhibited by free intracellular DHA in a dose-dependent manner [55], pointing to ALA as the superior substrate for DHA provision. ∆5 and ∆6 desaturase activities, encoded, respectively, by FADS1 / FADS2 genes, are recognized as main determinants of PUFA levels. Alterations of these enzymes and the presence of FADS1 /FADS2 gene polymorphisms have been associated with neuropsychiatric illnesses and neuroinflammation [56]. In addition, ALA dietary enrichment increases not only EPA and DHA but also the respective metabolic prostaglandins, thromboxanes, and leukotrienes, and the anti-inflammatory lipoxins and resolvins in various brain cells including neurons, astrocytes, and microglia [57], thus contributing to resistance against neuroinflammatory processes in AD [58]. The role of ALA metabolic enzymes in AD warrants additional research.

DHA supplementation has shown inconclusive results of both positive and negative effects related to BBB integrity [59] and improvement in AD symptoms [60]. This may be due to the inhibitory effects of DHA on ALA metabolism [55] and the lower formation of intermediate n-3 FA like EPA, also needed for membrane phospholipids building. Since ALA is the natural DHA precursor, and based on our previous knowledge of the beneficial effects of dietary ALA on metabolic disease [10,11], we propose that ALA may be an ideal source of brain DHA. The conversion of ALA to DHA and LA to AA proceeds via the same elongase and Δ6-desaturase enzymes and higher ALA intake levels may overcome the LA substrate competition, characteristic in Western diets, thus allowing the production of higher n-3 PUFA metabolites [61]. That is indeed what we found in C57Bl6/J mice, in which ALA dietary enrichment highly and significantly increased DHA levels in brain vasculature’s and brain parenchyma’s phospholipids, compared to control LA rich diets. Moreover, ALA enrichment also favored the higher Mfsd2a gene expression and protein levels [24] which may be correlated with the higher brain DHA levels. 

Saturated FA conversion into MUFA is regulated by ∆9 desaturase activity (SCD1 gene). Curiously, MUFA have been found, in some studies, to be elevated in AD patients brain, and to correlate with cognitive impairment, although the underlying mechanisms beyond this association are unknown [62]. Our recent studies have shown that ALA enrichment suppresses hepatic SCD1 mRNA expression and high fat diet–induced SCD1 increased activity [63] suggesting a potential benefit of dietary ALA enrichment by reducing the brain SCD1-mediated MUFA increase in AD.

In addition to the peripheral hepatic EFA metabolism and consequent brain transport, astrocytes have been suggested to be the central neural system providers of PUFA [64,65]. Surprisingly, based on our preliminary in vitro studies, human brain endothelial cells may be able to produce themselves DHA. The potential participation of cerebrovascular endothelial cells in FA metabolism seems to be corroborated by findings showing the protective role of long chain FA synthesis in angiogenesis [66]. This is important because it would imply the contribution of the brain endothelial cells in providing metabolically originated DHA to the brain throughout the lifespan.

## 8. Lifespan Fatty Acid Modifications

The early detection of AD-related brain lipid changes should be considered as a critical point related to the role of nutritional lipids in the prevention/restoration of the BBB during AD development. Based on our previous studies in C57Bl6/J mice [24], early age enrichment in dietary ALA significantly increases BBB phospholipids DHA content with beneficial effects on cognition at adult age. DHA deficiency in embryonic neurodevelopment can imprint long life brain damage, while AD pathology follows a long preclinical course, with DHA decrease being a hallmark of brain deterioration with aging [67]. The effect of DHA supplementation on the prevention or attenuation of AD symptoms have been inconsistent [68,69]. Surprisingly, ALA supplementation as a DHA precursor has not been similarly tested. This is probably due to the biased preconception that ALA is not efficiently transformed into DHA based on studies with a very high LA/ALA ratio which is characteristic of Western diets, and mainly on adult males. Pregnancy and fetal growth are, conversely, critical periods of neurodevelopment when the highest transformation of nutritional ALA into DHA supplies the offspring’s brain, with life-long impact [70,71]. Importantly, the ∆6 desaturase affinity for ALA decreases later than for LA [72] affirming ALA dietary enrichment as a reliable DHA source during the aging process. Therefore, based on existing results and due to the inhibitory hindrance of DHA on the ∆6 desaturase enzymes [55], we consider ALA as potentially optimal dietary substrate for DHA production for brain accretion, both in newborns and adults.

## 9. Cholesterol

Due to the BBB inherent impermeability, brain and peripheral cholesterol synthesis are separated [73]. Brain cholesterol levels are substantially reduced in ApoE4 AD patients compared with age-matched ApoE3 controls. The preferential degradation of ApoE4 relative to ApoE3 in astrocytes has been proposed to result in a reduced capacity for neuronal delivery of cholesterol, which may directly contribute to the disease progression [74]. The association between brain cholesterol levels and AD is still unclear. Recent work points to abnormalities in both cholesterol synthesis and catabolism in different patients’ brain areas [75]. Timely, our latest preliminary results (unpublished), show that the levels of brain and brain vasculature cholesterol in ApoE4 were reduced compared to those of ApoE3 Ki female mice, but significantly recovered by the ALA enriched diet. These results suggest that ALA may restore, at least partially, the extremely low cholesterol levels and thus, contribute to the BBB dysfunctional recovery.

## 10. ApoE4 and BBB Lipids in AD

Apolipoprotein E4 (ApoE4) allele carriers are at increased risk to develop AD compared with those carrying the ApoE3 or E2 alleles [76]. The ApoE4 genotype is the strongest Alzheimer’s susceptibility gene [77], and is associated with accelerated BBB breakdown and degeneration of brain capillary pericytes crucial for maintaining BBB integrity, reduced cerebral blood flow, and increased neuronal loss and cognitive decline in early and advanced stages of AD. Expression of the human ApoE4 gene in mice led to lower DHA transport across the BBB and lower proportion of DHA in brain when compared to ApoE2 expression [78]. These anomalies are independent of amyloid-β [1,9], insinuating that breakdown of the BBB contributes to ApoE4-associated cognitive decline independently of the AD classic pathology, and might be a therapeutic target in ApoE4 carriers [1]. The mechanisms responsible for the ApoE4 effects on BBB are unknown. Yet, the ApoE protein is a major cholesterol carrier that supports lipid transport and injury repair in the brain, suggesting deficient lipid transport as a plausible mechanism underlying its role in cognitive decline. Our emerging, yet unpublished results, suggest that ALA dietary enrichment in ApoE4 compared with ApoE3 mice brain, restores part of the decreased lipids, and in particular cholesterol and phospholipids, and leads to DHA enrichment in brain blood vessels, particularly in phosphatidylserine. These results suggest that BBB phospholipid disruption, is markedly restored by dietary ALA enrichment. Thus, altered lipid metabolism in brains of ApoE4 carriers may account for the barrier disintegration and consequent dysfunction, preceding other changes in brain function and behavior [79]. Interestingly, studies in a population with large-vessel disease show altered serum lipid profile in ApoE4 carriers, compared with ApoE2 and ApoE3 [80]. ApoE4 mice have shown increased susceptibility to endothelial cells lipid alterations [81], involving intracellular lipid flux and lipid droplet regulation as potential factors underlying higher AD risk [82]. These findings further support a role for lipid disarray in neurodegeneration and suggest nutritional modulation as a potentially efficacious therapeutic strategy in ApoE4 carriers [83]. Lower DHA has been consistently shown in ApoE4 AD mice brain [78]. Nevertheless, the few studies suggesting a link between FA and ApoE4 mostly refer to DHA supplements, and peripheral metabolism, but do not provide neither a clear mechanism nor a therapeutic target for the DHA effect on age associated neurodegeneration [84].

We predict that the link between ApoE4 genotype and AD evolution towards cognitive decline emerges, at least for some extent, from the BBB deterioration due to altered nutritional and biochemical FA processes leading to ineffective brain lipid accretion though the BBB.

## 11. Conclusions

**Figure 1:**
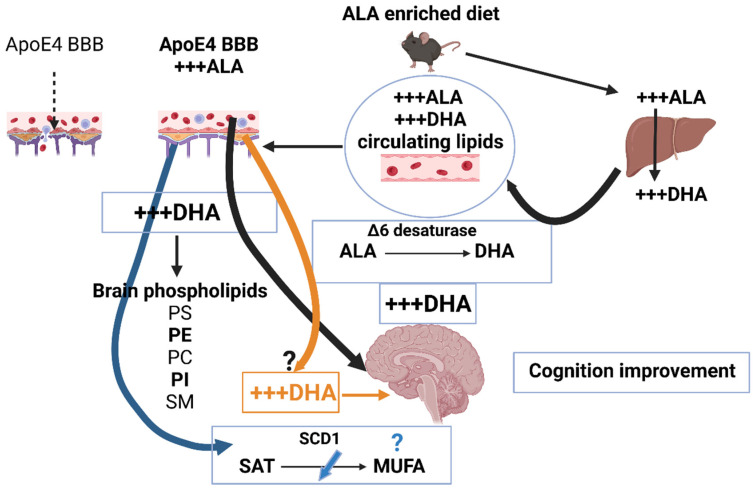
Conceptual model. This schematic picture illustrates how dietary fatty acids are ingested by the offspring, from the mother through the placenta, or by adult mice directly from the diet. Ingested FA are modified through the hepatic metabolism. High ALA levels increase DHA production through the pathway regulated by the key enzymatic ∆6 desaturase (black arrow on the right). Circulating FA reach the BBB. The decreased n-3 FA brain accretion, through Mfsd2a transporter expressed on brain endothelial cells, and dysfunctional BBB (left BBB cartoon) in AD are restored by ALA dietary enrichment (right BBB cartoon). Thus, increased DHA production rescues BBB function, brain accretion and phospholipids composition (left black arrow). These ALA induced modifications positively impact cognitive functioning. The potential desaturation of ALA to DHA by BBB may also contribute to brain DHA (orange arrow). The high SCD1 activity in AD on the transformation of Saturated to MUFA may be inhibited by ALA (blue arrow). Figures in this manuscript were created with Biorender.

The strong impact of ALA dietary enrichment on hepatic n-3 FA metabolism, may lead to restoration of DHA brain levels, by rescuing BBB disruption through membrane remodeling in brain endothelial cells. We envision that further research will unravel the mechanisms through which ALA enrichment improves BBB functionality, not only by repairing lipid transport, but also by enhancing brain ALA metabolism. In doing so, n-3 PUFA enrichment would comprise whole brain lipid homeostasis supporting restored cognitive functioning. This innovative concept opens the path for exciting prospects on ALA properties related to BBB in various brain diseases. Understanding the role and evolution of lipid modifications from early to old age, specifically in high risk ApoE4 carriers, would promote the discovery of novel nutritional strategies for the prevention, delay or restoration of cognitive decline. Figure 1 schematically illustrates this conceptual model.

## Data Availability

Not applicable.

## References

[1] Montagne A., Nation D.A., Sagare A.P., Barisano G., Sweeney M.D., Chakhoyan A., Pachicano M., Joe E., Nelson A.R., D’Orazio L.M. (2020). APOE4 leads to blood-brain barrier dysfunction predicting cognitive decline. Nature.

[2] Kadry H., Noorani B., Cucullo L. (2020). A blood-brain barrier overview on structure, function, impairment, and biomarkers of integrity. Fluids Barriers CNS.

[3] Ben-Zvi A., Lacoste B., Kur E., Andreone B.J., Mayshar Y., Yan H., Gu C. (2014). Mfsd2a is critical for the formation and function of the blood-brain barrier. Nature.

[4] Nguyen L.N., Ma D., Shui G., Wong P., Cazenave-Gassiot A., Zhang X., Wenk M.R., Goh E.L.K., Silver D.L. (2014). Mfsd2a is a transporter for the essential omega-3 fatty acid docosahexaenoic acid. Nature.

[5] Andreone B.J., Chow B.W., Tata A., Lacoste B., Ben-Zvi A., Bullock K., Deik A.A., Ginty D.D., Clish C.B., Gu C. (2017). Blood-Brain Barrier Permeability Is Regulated by Lipid Transport-Dependent Suppression of Caveolae-Mediated Transcytosis. Neuron.

[6] Rand D., Cooper I. (2021). Caspase-1: An important player and possible target for repair of the blood-brain barrier underlying neurodegeneration. Neural Regen. Res..

[7] Hung L., Levine H., Randhawa P., Park J. (2022). Technology-based group exercise interventions for people living with dementia or mild cognitive impairment: A scoping review protocol. BMJ Open.

[8] Cummings J., Lee G., Nahed P., Kambar M.E.Z.N., Zhong K., Fonseca J., Taghva K. (2022). Alzheimer’s disease drug development pipeline: 2022. Alzheimers Dement.

[9] Lotan R., Ganmore I., Livny A., Itzhaki N., Waserman M., Shelly S., Zacharia M., Moshier E., Uribarri J., Beisswenger P. (2021). Effect of Advanced Glycation End Products on Cognition in Older Adults with Type 2 Diabetes: Results from a Pilot Clinical Trial. J. Alzheimers Dis..

[10] Hollander K.S., Tempel Brami C., Konikoff F.M., Fainaru M., Leikin-Frenkel A. (2014). Dietary enrichment with alpha-linolenic acid during pregnancy attenuates insulin resistance in adult offspring in mice. Arch. Physiol. Biochem..

[11] Shomonov-Wagner L., Raz A., Leikin-Frenkel A. (2015). Alpha linolenic acid in maternal diet halts the lipid disarray due to saturated fatty acids in the liver of mice offspring at weaning. Lipids Health Dis..

[12] Elhaik Goldman S., Goez D., Last D., Naor S., Liraz Zaltsman S., Sharvit-Ginon I., Atrakchi-Baranes D., Shemesh C., Twitto-Greenberg R., Tsach S. (2018). High-fat diet protects the blood-brain barrier in an Alzheimer’s disease mouse model. Aging Cell.

[13] Gille B., Galuska C.E., Fuchs B., Peleg S. (2021). Recent Advances in Studying Age-Associated Lipids Alterations and Dietary Interventions in Mammals. Front. Aging.

[14] Cutuli D. (2017). Functional and Structural Benefits Induced by Omega-3 Polyunsaturated Fatty Acids During Aging. Curr. Neuropharmacol..

[15] Bowman G.L., Dodge H.H., Guyonnet S., Zhou N., Donohue J., Bichsel A., Schmitt J., Hooper C., Bartfai T., Andrieu S. (2019). A blood-based nutritional risk index explains cognitive enhancement and decline in the multidomain Alzheimer prevention trial. Alzheimers Dement.

[16] Norwitz N.G., Saif N., Ariza I.E., Isaacson R.S. (2021). Precision nutrition for alzheimer’s prevention in apoe4 carriers. Nutrients.

[17] Yassine H.N., Samieri C., Livingston G., Glass K., Wagner M., Tangney C., Plassman B.L., Ikram M.A., Voigt R.M., Gu Y. (2022). Nutrition state of science and dementia prevention: Recommendations of the Nutrition for Dementia Prevention Working Group. Lancet Healthy Longev..

[18] Abbott N.J., Patabendige A.A.K., Dolman D.E.M., Yusof S.R., Begley D.J. (2010). Structure and function of the blood-brain barrier. Neurobiol. Dis..

[19] Epand R.M. (2007). Membrane lipid polymorphism: Relationship to bilayer properties and protein function. Methods Mol. Biol..

[20] Mukerjee S., Saeedan A.S., Ansari M.N., Singh M. (2021). Polyunsaturated fatty acids mediated regulation of membrane biochemistry and tumor cell membrane integrity. Membranes.

[21] Dyall S.C. (2015). Long-chain omega-3 fatty acids and the brain: A review of the independent and shared effects of EPA, DPA and DHA. Front. Aging Neurosci..

[22] Gimenez M.S., Oliveros L.B., Gomez N.N. (2011). Nutritional deficiencies and phospholipid metabolism. Int. J. Mol. Sci..

[23] Escribá P.V. (2006). Membrane-lipid therapy: A new approach in molecular medicine. Trends Mol. Med..

[24] Leikin-Frenkel A., Liraz-Zaltsman S., Hollander K.S., Atrakchi D., Ravid O., Rand D., Kandel-Kfir M., Israelov H., Cohen H., Kamari Y. (2021). Dietary alpha linolenic acid in pregnant mice and during weaning increases brain docosahexaenoic acid and improves recognition memory in the offspring. J. Nutr. Biochem..

[25] Montagne A., Zhao Z., Zlokovic B.V. (2017). Alzheimer’s disease: A matter of blood-brain barrier dysfunction?. J. Exp. Med..

[26] Chang C.-Y., Ke D.-S., Chen J.-Y. (2009). Essential fatty acids and human brain. Acta Neurol. Taiwan.

[27] Bazan N.G., Musto A.E., Knott E.J. (2011). Endogenous signaling by omega-3 docosahexaenoic acid-derived mediators sustains homeostatic synaptic and circuitry integrity. Mol. Neurobiol..

[28] Svennerholm L., Boström K., Jungbjer B. (1997). Changes in weight and compositions of major membrane components of human brain during the span of adult human life of Swedes. Acta Neuropathol..

[29] Eelen G., de Zeeuw P., Treps L., Harjes U., Wong B.W., Carmeliet P. (2018). Endothelial Cell Metabolism. Physiol. Rev..

[30] Sampath H., Ntambi J.M. (2005). Polyunsaturated fatty acid regulation of genes of lipid metabolism. Annu. Rev. Nutr..

[31] Söderberg M., Edlund C., Kristensson K., Dallner G. (1991). Fatty acid composition of brain phospholipids in aging and in Alzheimer’s disease. Lipids.

[32] Hennebelle M., Plourde M., Chouinard-Watkins R., Castellano C.-A., Barberger-Gateau P., Cunnane S.C. (2014). Ageing and apoE change DHA homeostasis: Relevance to age-related cognitive decline. Proc. Nutr. Soc..

[33] Kurz C., Walker L., Rauchmann B.-S., Perneczky R. (2022). Dysfunction of the blood-brain barrier in Alzheimer’s disease: Evidence from human studies. Neuropathol. Appl. Neurobiol..

[34] Sweeney M.D., Sagare A.P., Zlokovic B.V. (2018). Blood-brain barrier breakdown in Alzheimer disease and other neurodegenerative disorders. Nat. Rev. Neurol..

[35] Cullis P.R., de Kruijff B. (1979). Lipid polymorphism and the functional roles of lipids in biological membranes. Biochim. Biophys. Acta.

[36] Cai Z., Qiao P.-F., Wan C.-Q., Cai M., Zhou N.-K., Li Q. (2018). Role of Blood-Brain Barrier in Alzheimer’s Disease. J. Alzheimers Dis..

[37] Barnes S., Chowdhury S., Gatto N.M., Fraser G.E., Lee G.J. (2021). Omega-3 fatty acids are associated with blood-brain barrier integrity in a healthy aging population. Brain Behav..

[38] Chang C.-Y., Kuan Y.-H., Li J.-R., Chen W.-Y., Ou Y.-C., Pan H.-C., Liao S.-L., Raung S.-L., Chang C.-J., Chen C.-J. (2013). Docosahexaenoic acid reduces cellular inflammatory response following permanent focal cerebral ischemia in rats. J. Nutr. Biochem..

[39] Kuo Y.-T., So P.-W., Parkinson J.R., Yu W.S., Hankir M., Herlihy A.H., Goldstone A.P., Frost G.S., Wasserfall C., Bell J.D. (2010). The combined effects on neuronal activation and blood-brain barrier permeability of time and n-3 polyunsaturated fatty acids in mice, as measured in vivo using MEMRI. Neuroimage.

[40] Hooijmans C.R., Pasker-de Jong P.C.M., de Vries R.B.M., Ritskes-Hoitinga M. (2012). The effects of long-term omega-3 fatty acid supplementation on cognition and Alzheimer’s pathology in animal models of Alzheimer’s disease: A systematic review and meta-analysis. J. Alzheimers Dis..

[41] Yamazaki Y., Shinohara M., Shinohara M., Yamazaki A., Murray M.E., Liesinger A.M., Heckman M.G., Lesser E.R., Parisi J.E., Petersen R.C. (2019). Selective loss of cortical endothelial tight junction proteins during Alzheimer’s disease progression. Brain.

[42] Ntambi J.M., Bené H. (2001). Polyunsaturated fatty acid regulation of gene expression. J. Mol. Neurosci..

[43] Cho H.P., Nakamura M., Clarke S.D. (1999). Cloning, expression, and fatty acid regulation of the human delta-5 desaturase. J. Biol. Chem..

[44] McNamara R.K., Liu Y., Jandacek R., Rider T., Tso P. (2008). The aging human orbitofrontal cortex: Decreasing polyunsaturated fatty acid composition and associated increases in lipogenic gene expression and stearoyl-CoA desaturase activity. Prostaglandins Leukot Essent Fatty Acids.

[45] Bazan N.G. (2003). Synaptic lipid signaling: Significance of polyunsaturated fatty acids and platelet-activating factor. J. Lipid Res..

[46] Katsuki H., Okuda S. (1995). Arachidonic acid as a neurotoxic and neurotrophic substance. Prog. Neurobiol..

[47] Wood C.A.P., Zhang J., Aydin D., Xu Y., Andreone B.J., Langen U.H., Dror R.O., Gu C., Feng L. (2021). Structure and mechanism of blood-brain-barrier lipid transporter MFSD2A. Nature.

[48] Leikin A., Shinitzky M. (1995). Characterization of the lipid surrounding the delta 6-desaturase of rat liver microsomes. Biochim. Biophys. Acta.

[49] Dyall S.C., Michael-Titus A.T. (2008). Neurological benefits of omega-3 fatty acids. Neuromolecul. Med..

[50] Von Schacky C. (2021). Importance of EPA and DHA blood levels in brain structure and function. Nutrients.

[51] Liu J.-H., Wang Q., You Q.-L., Li Z.-L., Hu N.-Y., Wang Y., Jin Z.-L., Li S.-J., Li X.-W., Yang J.-M. (2020). Acute EPA-induced learning and memory impairment in mice is prevented by DHA. Nat. Commun..

[52] Bradbury J. (2011). Docosahexaenoic acid (DHA): An ancient nutrient for the modern human brain. Nutrients.

[53] Du Q., Martin J.-C., Agnani G., Pages N., Leruyet P., Carayon P., Delplanque B. (2012). Dairy fat blends high in α-linolenic acid are superior to n-3 fatty-acid-enriched palm oil blends for increasing DHA levels in the brains of young rats. J. Nutr. Biochem..

[54] Tu W.C., Cook-Johnson R.J., James M.J., Mühlhäusler B.S., Gibson R.A. (2010). Omega-3 long chain fatty acid synthesis is regulated more by substrate levels than gene expression. Prostaglandins Leukot Essent Fatty Acids.

[55] Majou D. (2021). Synthesis of DHA (omega-3 fatty acid): *FADS2* gene polymorphisms and regulation by PPARα. OCL.

[56] Tosi F., Sartori F., Guarini P., Olivieri O., Martinelli N. (2014). Delta-5 and delta-6 desaturases: Crucial enzymes in polyunsaturated fatty acid-related pathways with pleiotropic influences in health and disease. Adv. Exp. Med. Biol..

[57] Das U.N. (2006). Essential fatty acids: Biochemistry, physiology and pathology. Biotechnol. J..

[58] Akiyama H., Barger S., Barnum S., Bradt B., Bauer J., Cole G.M., Cooper N.R., Eikelenboom P., Emmerling M., Fiebich B.L. (2000). Inflammation and Alzheimer’s disease. Neurobiol. Aging.

[59] Lindenau K.L., Barr J.L., Higgins C.R., Sporici K.T., Brailoiu E., Brailoiu G.C. (2022). Blood-Brain Barrier Disruption Mediated by FFA1 Receptor-Evidence Using Miniscope. Int. J. Mol. Sci..

[60] Heath R.J., Wood T.R. (2021). Why have the benefits of DHA not been borne out in the treatment and prevention of alzheimer’s disease? A narrative review focused on DHA metabolism and adipose tissue. Int. J. Mol. Sci..

[61] Brenner R.R. (1974). The oxidative desaturation of unsaturated fatty acids in animals. Mol. Cell. Biochem..

[62] Astarita G., Jung K.-M., Vasilevko V., Dipatrizio N.V., Martin S.K., Cribbs D.H., Head E., Cotman C.W., Piomelli D. (2011). Elevated stearoyl-CoA desaturase in brains of patients with Alzheimer’s disease. PLoS ONE.

[63] Leikin-Frenkel A., Cohen H., Keshet R., Shnerb-GanOr R., Kandel-Kfir M., Harari A., Hollander K.S., Shaish A., Harats D., Kamari Y. (2022). The effect of α-linolenic acid enrichment in perinatal diets in preventing high fat diet-induced SCD1 increased activity and lipid disarray in adult offspring of low density lipoprotein receptor knockout (LDLRKO) mice. Prostaglandins Leukot. Essent. Fatty Acids.

[64] Bernoud N., Fenart L., Bénistant C., Pageaux J.F., Dehouck M.P., Molière P., Lagarde M., Cecchelli R., Lecerf J. (1998). Astrocytes are mainly responsible for the polyunsaturated fatty acid enrichment in blood-brain barrier endothelial cells in vitro. J. Lipid Res..

[65] Taylor X., Cisternas P., Jury N., Martinez P., Huang X., You Y., Redding-Ochoa J., Vidal R., Zhang J., Troncoso J. (2022). Activated endothelial cells induce a distinct type of astrocytic reactivity. Commun. Biol..

[66] Wang J., Xu J., Zang G., Zhang T., Wu Q., Zhang H., Chen Y., Wang Y., Qin W., Zhao S. (2022). trans-2-Enoyl-CoA Reductase Tecr-Driven Lipid Metabolism in Endothelial Cells Protects against Transcytosis to Maintain Blood-Brain Barrier Homeostasis. Research.

[67] Martinat M., Rossitto M., Di Miceli M., Layé S. (2021). Perinatal dietary polyunsaturated fatty acids in brain development, role in neurodevelopmental disorders. Nutrients.

[68] Phillips M.A., Childs C.E., Calder P.C., Rogers P.J. (2015). No Effect of Omega-3 Fatty Acid Supplementation on Cognition and Mood in Individuals with Cognitive Impairment and Probable Alzheimer’s Disease: A Randomised Controlled Trial. Int. J. Mol. Sci..

[69] Quinn J.F., Raman R., Thomas R.G., Yurko-Mauro K., Nelson E.B., Van Dyck C., Galvin J.E., Emond J., Jack C.R., Weiner M. (2010). Docosahexaenoic acid supplementation and cognitive decline in Alzheimer disease: A randomized trial. JAMA.

[70] Burdge G.C., Calder P.C. (2005). Conversion of alpha-linolenic acid to longer-chain polyunsaturated fatty acids in human adults. Reprod. Nutr. Dev..

[71] Burdge G. (2004). Alpha-linolenic acid metabolism in men and women: Nutritional and biological implications. Curr. Opin. Clin. Nutr. Metab. Care.

[72] Hrelia S., Bordoni A., Celadon M., Turchetto E., Biagi P.L., Rossi C.A. (1989). Age-related changes in linoleate and alpha-linolenate desaturation by rat liver microsomes. Biochem. Biophys. Res. Commun..

[73] Björkhem I., Meaney S. (2004). Brain cholesterol: Long secret life behind a barrier. Arterioscler. Thromb. Vasc. Biol..

[74] Riddell D.R., Zhou H., Atchison K., Warwick H.K., Atkinson P.J., Jefferson J., Xu L., Aschmies S., Kirksey Y., Hu Y. (2008). Impact of apolipoprotein E (ApoE) polymorphism on brain ApoE levels. J. Neurosci..

[75] Varma V.R., Büşra Lüleci H., Oommen A.M., Varma S., Blackshear C.T., Griswold M.E., An Y., Roberts J.A., O’Brien R., Pletnikova O. (2021). Abnormal brain cholesterol homeostasis in Alzheimer’s disease-a targeted metabolomic and transcriptomic study. Npj Aging Mech. Dis..

[76] Liu C.-C., Liu C.-C., Kanekiyo T., Xu H., Bu G. (2013). Apolipoprotein E and Alzheimer disease: Risk, mechanisms and therapy. Nat. Rev. Neurol..

[77] Corder E.H., Saunders A.M., Strittmatter W.J., Schmechel D.E., Gaskell P.C., Small G.W., Roses A.D., Haines J.L., Pericak-Vance M.A. (1993). Gene dose of apolipoprotein E type 4 allele and the risk of Alzheimer’s disease in late onset families. Science.

[78] Vandal M., Alata W., Tremblay C., Rioux-Perreault C., Salem N., Calon F., Plourde M. (2014). Reduction in DHA transport to the brain of mice expressing human APOE4 compared to APOE2. J. Neurochem..

[79] Barisano G., Kisler K., Wilkinson B., Nikolakopoulou A.M., Sagare A.P., Wang Y., Gilliam W., Huuskonen M.T., Hung S.-T., Ichida J.K. (2022). A “multi-omics” analysis of blood-brain barrier and synaptic dysfunction in APOE4 mice. J. Exp. Med..

[80] Saidi S., Slamia L.B., Ammou S.B., Mahjoub T., Almawi W.Y. (2007). Association of apolipoprotein E gene polymorphism with ischemic stroke involving large-vessel disease and its relation to serum lipid levels. J. Stroke Cerebrovasc. Dis..

[81] Miranda A.M., Ashok A., Chan R.B., Zhou B., Xu Y., McIntire L.B., Area-Gomez E., Di Paolo G., Duff K.E., Oliveira T.G. (2022). Effects of APOE4 allelic dosage on lipidomic signatures in the entorhinal cortex of aged mice. Transl. Psychiatry.

[82] Qi G., Mi Y., Shi X., Gu H., Brinton R.D., Yin F. (2021). ApoE4 Impairs Neuron-Astrocyte Coupling of Fatty Acid Metabolism. Cell Rep..

[83] Mallick R., Duttaroy A.K. (2022). Modulation of endothelium function by fatty acids. Mol. Cell. Biochem..

[84] Yin F. (2022). Lipid metabolism and Alzheimer’s disease: Clinical evidence, mechanistic link and therapeutic promise. FEBS J..

